# Effect of Thermal Exposure on Residual Properties of Wet Layup Carbon Fiber Reinforced Epoxy Composites

**DOI:** 10.3390/polym14142957

**Published:** 2022-07-21

**Authors:** SoonKook Hong, Vistasp M. Karbhari

**Affiliations:** 1 Department of Mechanical and Naval Architectural Engineering, Naval Academy, Changwon City 440-746, Korea; hsk753@mnd.go.kr; 2 Department of Civil Engineering, and Department of Mechanical & Aerospace Engineering, University of Texas Arlington, Arlington, TX 76019, USA

**Keywords:** polymer matrix composites, thermal exposure, residual characteristics, mechanical properties, deterioration

## Abstract

Ambient cured wet layup carbon fiber reinforced epoxy composites used extensively in the rehabilitation of infrastructure and in structural components can be exposed to elevated temperature regimes for extended periods of time of hours to a few days due to thermal excursions. These may be severe enough to cause a significant temperature rise without deep charring as through fires at a small distance and even high-temperature industrial processes. In such cases, it is critical to have information related to the post-event residual mechanical properties and damage states. In this paper, composites are subjected to a range of elevated temperatures up to 260 °C over periods of time up to 72 h. Exposure to elevated temperature regimes is noted to result in a competition between the mechanisms of post-cure that can increase the levels of mechanical characteristics, and the deterioration of the resin and the bond between the fibers and resin that can reduce them. Mechanical tests indicate that tensile and short beam shear properties are not affected negatively until the highest temperatures of exposure considered in this investigation. In contrast, all elevated temperature conditions cause deterioration in resin-dominated characteristics such as shear and flexure, emphasizing the weakness of this mode in layered composites formed from unidirectional fabric architectures due to resin deterioration. Transitions in failure modes are correlated through microscopy to damage progression both at the level of fiber-matrix interface integrity and through the bulk resin, especially at the inter-layer level. The changes in glass transition temperature determined through differential scanning calorimetry can be related to thresholds that indicate changes in the mechanisms of damage.

## 1. Introduction

Carbon fiber reinforced polymer (CFRP) composites are used extensively for the rehabilitation of deteriorating and understrength infrastructure components in bridges, buildings, and industrial facilities, as well as in naval infrastructure including jetties, piers, pilings, and shipboard structural components due to their light weight, high specific properties, tailorability, ease of placement in the field, and potentially high durability. In these applications, the composites are manufactured primarily using pultrusion, resin infusion, and wet layup-type processes. The wet layup process enables flexibility and ease of placement in the field and is hence used extensively for the rehabilitation of concrete structures through external bonding. However, the use of a manual process for placement and cure under ambient conditions results in lower fiber volume fractions, higher void content, lower levels of glass transition temperature, and slow progression to full polymerization and cure [1] as compared to the autoclave-cured aerospace composites and even those fabricated by pultrusion and resin infusion.

Carbon fibers are largely unaffected by general environmental exposure conditions likely to be faced in these applications, although they do oxidize at temperatures exceeding 500 °C [2,3]. In contrast, epoxy resins and the bond between the fiber and the resin can be affected by a range of exposures [4] resulting in degradation-based changes in mechanical performance characteristics. In particular, exposure to elevated temperatures is known to cause the degradation of the polymer, and consequently of the composite as well, when exposed to temperature levels at, or above, the glass transition temperature [5]. Composite materials are known to degrade through ablation and charring which causes loss of material and deterioration in performance because of thermal aging, even prior to material loss [6]. Notwithstanding the higher temperature resistance of reinforcing fibers, polymer matrix composites are susceptible to thermo-oxidative degradation at temperatures between 100 °C and 350 °C, with effects increasing in the vicinity of, and above, the glass transition temperature. The effects include those of physical aging which are reversible, accruing from the decreased molecular mobility of the polymer network resulting in the increased evolution of strain and energy. As temperature levels and time of exposure are increased, irreversible volumetric-based chemical aging occurs, which includes chain scission reactions and additional cross-linking, both of which can cause changes to surface diffusion response. Thermal aging in air can lead to superficial oxidation which leads to cracking without the application of load [7,8,9], which can further the diffusion of oxygen into the material, resulting in additional deeper oxidation reactions and damage [10]. The yield strength of the resin and the interphase bond between the resin and fiber decrease with an increase in temperature [11], with shear stiffness and interfacial shear strength decreasing as a function of temperature in epoxy-based composites [11,12]. Wimolkiatisak and Bell [13] further noted that the stiffness of the resin was reduced initially by exposure to elevated temperatures giving fibers freedom to move which could result in further degradation of even fiber-dominated characteristics due to fiber misalignment. The importance of these phenomena on the stability and durability of polymer composites in the civil, marine, and naval areas has resulted in studies of composites exposed to fires [2,14,15,16,17,18]. While a significant body of work exists related to autoclave-cured aerospace-grade carbon fiber reinforced composites, significantly less is known about the response of materials fabricated through wet layup and non-autoclave cure processes [19] and especially related to the competing effects of the initial cure progression and the deteriorative effects of elevated temperatures where the effects are due to radiant heat rather than actual contact with flames. It is critical to understand the materials level processes of deterioration and characterize the post-thermal exposure residual performance attributes of composites that are routine, as in the case of industrial processes or gas flares, or on occasion, exposed to elevated temperature regimes for extended periods of time, from a few hours to a few days [16,20]. Given that many of the structural components and structural elements, including those used in the rehabilitation of concrete infrastructure even relatively far from the actual flame and/or locus of heat, could face the conditions of thermal aging due to elevated temperature exposure, there is a need to assess the change in the performance characteristics and especially residual properties after exposure [21]. In general, it is essential to be able to characterize the residual structural integrity and safety post-exposure, and at present, there is a lack of a substantial body of data in this area [22].

The performance characteristics of epoxies and composites during and after exposure to thermal excursions are dependent on the temperature level, polymer morphology and constituents [4,23], and the degree of cure and crosslink density [24], among other factors. Mechanisms associated with the degradation of the epoxies of the type used in such applications have been discussed by Burton [25], Grassie [26], and Levchik [27]. Reviews of the degradation of composites exposed to fire and elevated temperatures, especially of glass fiber-reinforced polymer composites, have been reported by Firmo et al. [16], Singh and Sethi [17], and Bazli and Abolfazli [18], among others. In addition, a substantial body of work exists based on the investigation of naval and marine composites exposed to fire and thermal excursions which share many of the characteristics of those used in civil infrastructure rehabilitation exposed to fire [28,29,30]. In a series of studies, Sorathia et al. [31,32,33] showed that composites with a thickness of about 5 mm can lose more than 75% of their flexural strength as a result of a 20-min exposure to a low to medium intensity fire characterized by heat flux of 25 kW/m^2^. Moritz and Mathys [34,35] reported large reductions in the post-fire tension, compression, flexure, and interlaminar shear properties of glass fiber-reinforced polyester composites. More recently Nguyen et al. reported on a series of tests on pultruded and wet- or hand-layup CFRP under combined thermal and mechanical loading showing that strength loss can be greater than 50%, with modulus loss being relatively lower, with a decrease of 30% at 600 °C [36,37,38], and that exposure temperatures required to attain a prespecified loss threshold could be affected by the level of imposed load [38]. A majority of these and other studies, especially those conducted on marine composites, relate to fire damage that results from the actual ignition of the polymer and the charring or burning of the composite. While this is extremely relevant when the composite is itself engulfed in the fire, it does not provide the information of use for the design and assessment of composites that could be routinely, or on occasion, exposed to elevated temperature regimes for extended periods of time of hours to a few days due to thermal excursions that may be severe enough to cause a significant temperature rise without deep charring as through fires at a small distance and even high-temperature industrial processes. There is thus a substantial need to better characterize and understand the post-thermal exposure residual properties of these composites [16,19,20,39,40,41].

The current study is part of a larger ongoing investigation into residual and thermal characteristics of wet layup CFRP and is focused on further understanding the effects of thermal aging on damage progression and post-thermal exposure mechanical characteristics. The selected system is representative of materials used in infrastructure rehabilitation. This study focuses on a range of temperatures between 23 °C and 260 °C, with exposure periods up to 72 h at temperature prior to testing for residual properties. The temperature range corresponds to that likely to be seen in the field, especially for cases where the composite is not in contact with the flame but could be exposed to levels of elevated temperature for extended periods of time and matches that used by Zavatta et al. at the higher level [42], and near, but below, the resin decomposition temperature [43]. It is also within the range likely to be felt by the composite that was protected by a layer of insulation that separated it from the fire. Residual characteristics are determined through tensile, off-axis shear, flexure, and short beam shear tests conducted after exposure at temperature for specified periods of time. Scanning electron microscopy and differential scanning calorimetry are also used to assess the damage/deterioration and changes in glass transition temperature as a means of further understanding materials’ level effects and transitions.

## 2. Experimental Program

### 2.1. Material System

The CFRP composite system used consisted of a unidirectional carbon fabric of an aerial weight of 644 g/m^2^ and a difunctional Bisphenol A/epichlorohydrin-derived liquid epoxy (Epon 828) with an epoxide equivalent weight of 185–192 g/cc and a number average molecular weight of about 700. A polyetheramine-modified polyoxypropylenediamine curing agent with an average molecular weight of about 400 was used with a system viscosity of 600–700 cps at 25 °C. The carbon fibers had a nominal tensile strength and modulus of 3.79 GPa and 230 GPa, respectively, and the neat resin has a tensile strength and modulus of 70 MPa and 3 GPa, respectively. Two-layer composites were fabricated using the wet layup process, replicating procedures used in the field to mimic actual rehabilitation conditions as closely as possible. Panels of unidirectional and 0/90 layups (used to form ±45 specimens for off-axis shear testing) were fabricated and allowed to cure under control conditions of 23 °C and 30% relative humidity for seven days, after which they were post-cured for 72 h at 60 °C. The fiber weight fraction was determined using acid digestion procedures to be 60% (i.e., a fiber volume fraction of 49.8%) with a standard deviation of 2–2.5% over all specimens.

### 2.2. Test Procedures

To assess the effects of exposure to elevated temperature on residual mechanical properties, tests were conducted in tension, flexure, off-axis shear, and short beam shear modes. The tensile tests were conducted on unidirectional specimens in the fiber direction following ASTM D3039 on specimens of 254 mm in length and 12.7 mm in width, with a gauge length of 155 mm at a displacement rate of 1.27 mm/min. using an extensometer to measure strains. The flexure tests were conducted in three-point bend mode in accordance with ASTM D790 on unidirectional specimens with fibers along the span with a width of 12.7 mm and a span to depth ratio of 16:1. For the purposes of the current investigation flexural strength was determined by

(1) $$\sigma_{f}=\frac{3Pl}{2bh^{2}}$$

where *P* is the load and *b*, *l*, and *h* are the specimen width, span, and thickness, respectively. The flexural strain was defined as the nominal fractional change in the length of an element of the outer surface at midspan and was determined as

(2) $$\varepsilon_{f}=\frac{6\delta h}{l^{2}}$$

where *δ* is the maximum deflection measured at the center span of the beam. The flexural modulus is represented through the chord modulus which was determined from the load deflection curve as

(3) $$E_{f}=\frac{\left(\sigma_{f2}-\sigma_{f1}\right)}{\left(\epsilon_{f2}-\varepsilon_{f1}\right)}$$

where the subscripts 1 and 2 represent discrete points. Loading was at a crosshead speed of 2 mm/min. Off-axis shear tests were conducted following ASTM D3518 with specimens cut from the 0/90 panels to acquire ±45 orientations in the gauge length of 140 mm. Short beam shear (SBS) tests were conducted to assess interlaminar shear strength using unidirectional specimens following ASTM D2344 with a width of 6 mm and length to thickness of 6:1 at a crosshead speed of 1 mm/min, with short beam shear (SBS) strength determined as

(4) $$\sigma_{\mathrm{SBS}}=\frac{0.75\;P}{bh}$$

where *P* is the failure load. Five specimens were tested for each condition for all tests except the off-axis shear tests, for which three specimens were used. All specimens were cut to size and then stored in a humidity chamber at 23 °C and 30% relative humidity for two weeks prior to testing. In addition to tests conducted under ambient conditions as a baseline, specimens were exposed to temperatures between 150 °F and 500 °F at intervals of 50 °F i.e., at 66 °C, 93 °C, 121 °C, 149 °C, 177 °C, 204 °C, 232 °C, and 260 °C for periods of time ranging from 1 to 72 h. In each case, the specimens were placed in a furnace that was already at temperature and were tested after removal and cooling back to 23 °C to acquire residual characteristics.

To characterize the effects of elevated temperature exposure on the cure and glass transition temperature (T_g_) of the materials, tests were also conducted using differential scanning calorimetry (DSC) following ASTM D3418 on specimens of 10–15 mg heated at a rate of 10 °C/min from 0 to the final temperature of 160 °C in a controlled nitrogen environment with a flow rate of 10 mL/min. Liquid nitrogen gas was used for the initial setting of 0 °C.

Scanning electron microscopy (SEM) was used to assess cross-sections of the specimens for deterioration focusing on resin and fiber-matrix bond characteristics.

## 3. Results and Discussion

The exposure of wet layup, ambient-cured composites to elevated temperatures results in a competition between the mechanisms of post-cure, which is likely to result in an increase in some mechanical characteristics, and those of deterioration due to temperature. In terms of the latter, it should be noted that the regimes selected do not cross the threshold for the deterioration of carbon fibers, which is about 500 °C for the initiation of oxidation, and hence the deterioration would be restricted to the resin and the fiber-matrix interphase.

### 3.1. Mechanical Characterization

#### 3.1.1. Uniaxial Tension

**Figure 1:**
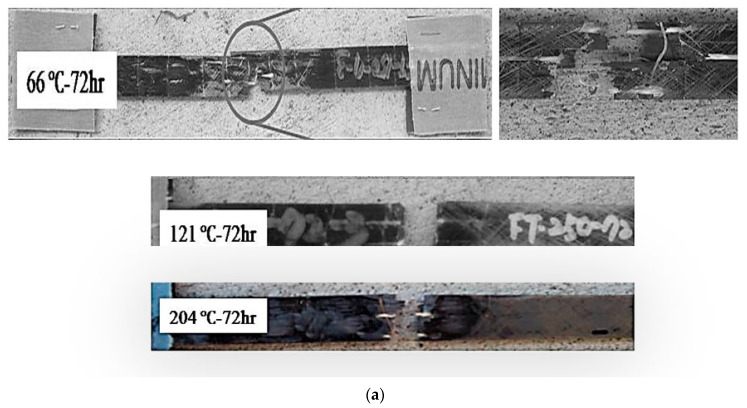

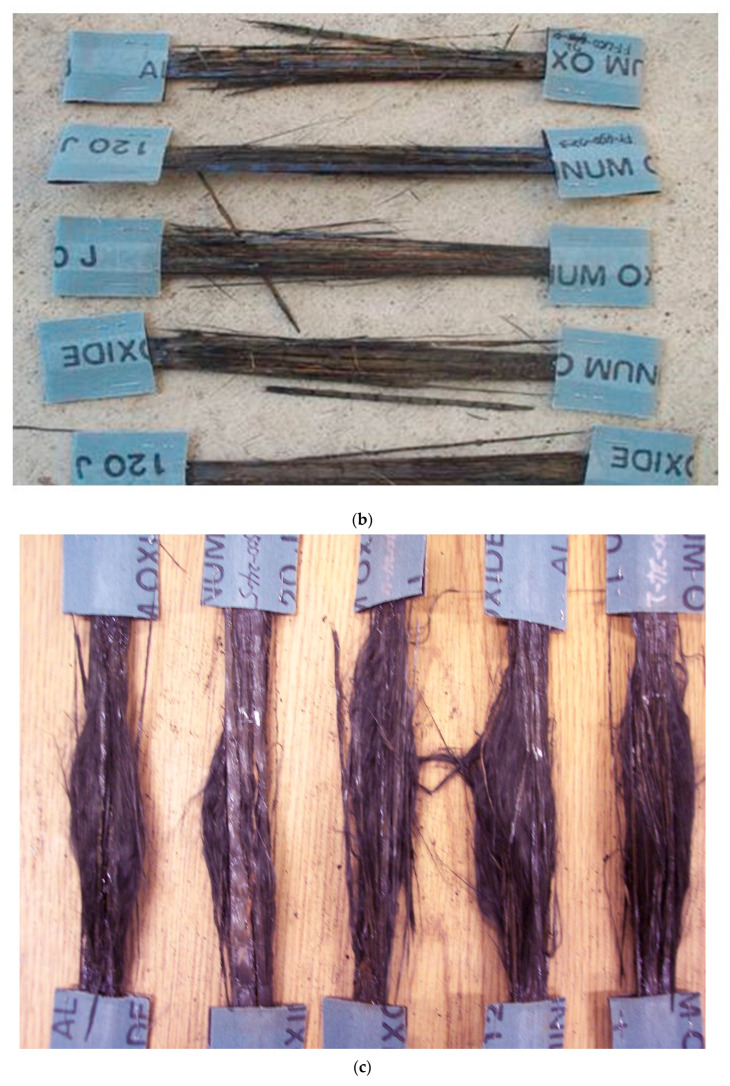
(**a**) Characteristic tensile failure within the gauge length. The figure on the top right shows a closeup of fracture surfaces; (**b**) Longitudinal splitting failure after exposure at 232 °C; (**c**) Brooming mode of failure due to resin degradation after exposure at 260 °C.

**Figure 2:**
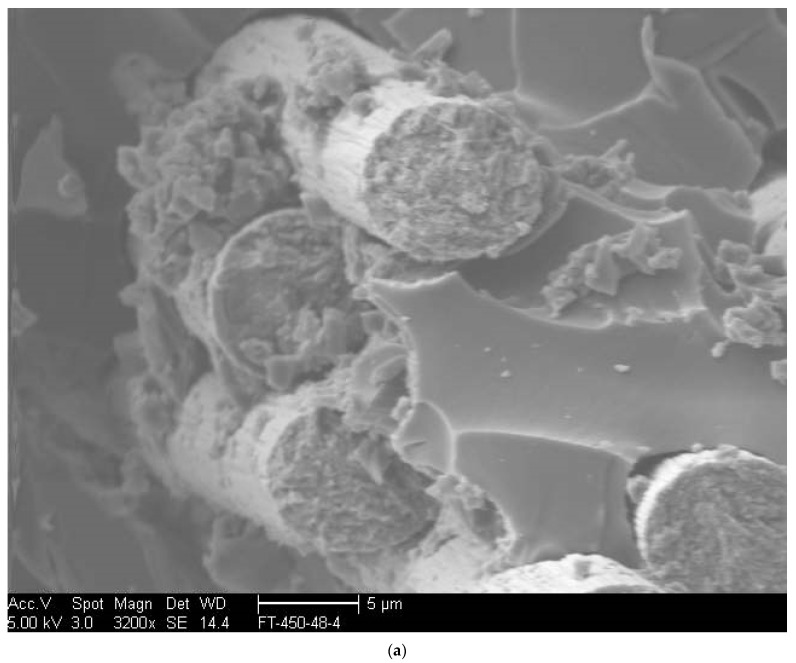

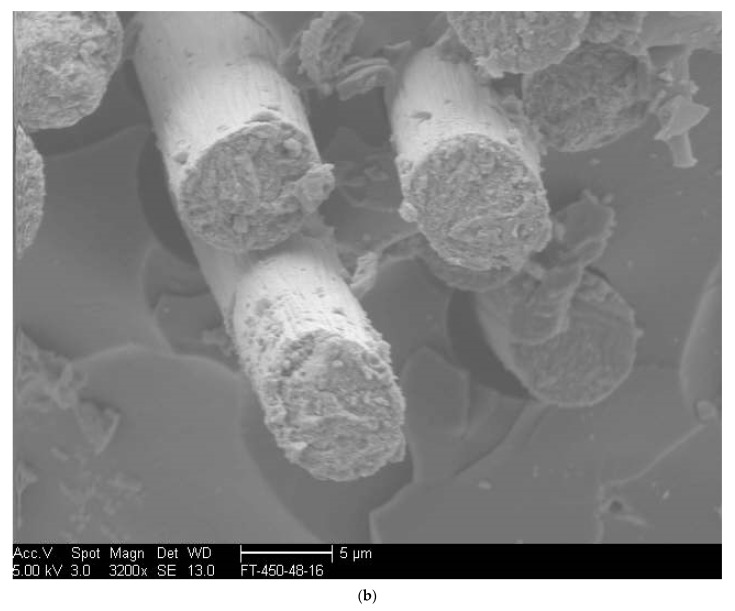

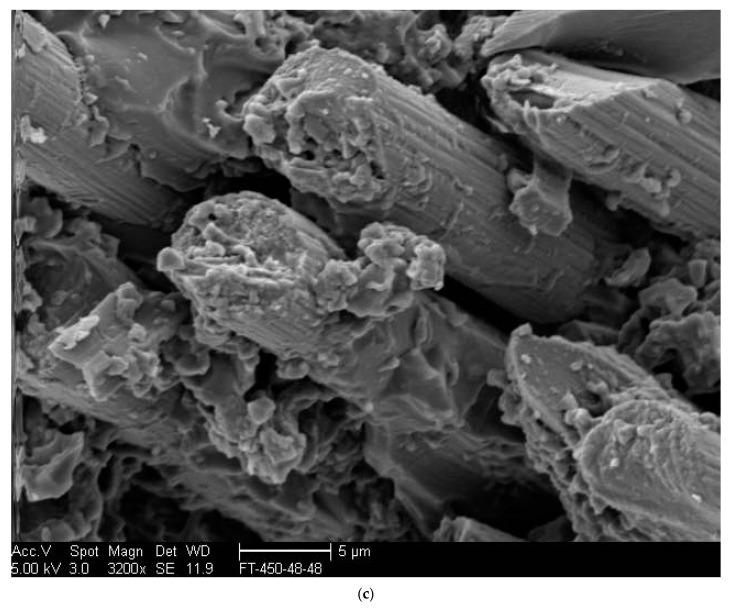
(**a**) Scanning Electron Micrograph of specimen after 4 h of exposure at 232 °C; (**b**) Scanning Electron Micrograph of specimen after 16 h of exposure at 232 °C; (**c**) Scanning Electron Micrograph of specimen after 48 h of exposure at 232 °C.

**Figure 3:**
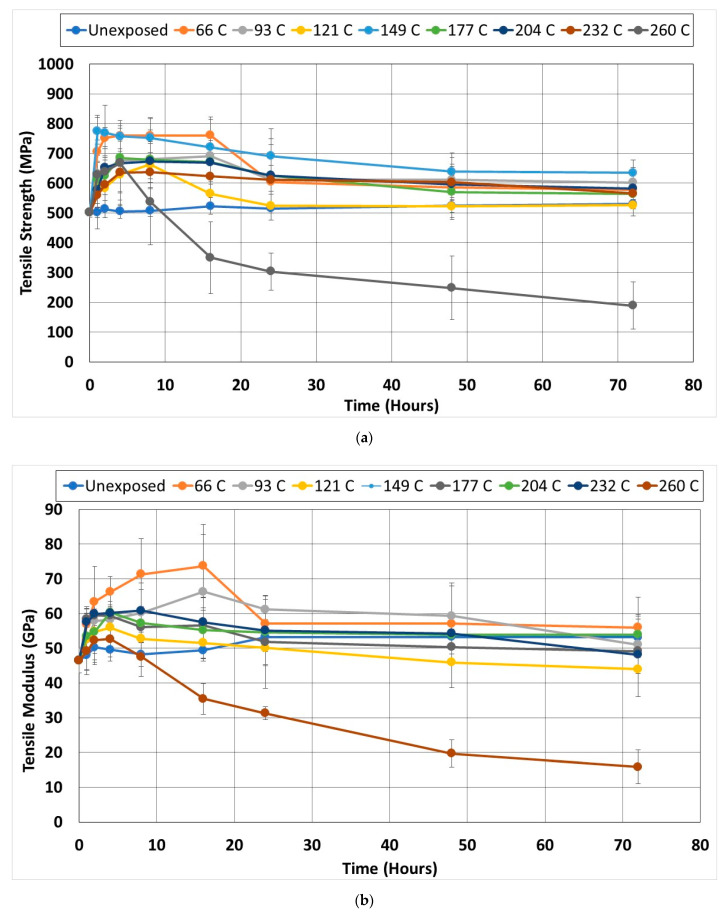
(**a**) Change in tensile strength as a function of exposure temperature and time; (**b**) Change in Tensile Modulus as a function of exposure temperature and time.

As seen in Figure 1a, specimens exposed to temperature levels up to 204 °C failed through characteristic fiber fracture-based tensile failure modes within the gauge length of the specimens. In contrast, at 232 °C the failure mechanism is one of longitudinal splitting (Figure 1b) driven by the initiation of the degradation of the bond between the fiber and the matrix. This is similar to the mode reported by Hawileh et al. [44] for specimens exposed to temperatures between 200 and 250 °C and was attributed to the partial loss of epoxy. At the highest temperature of exposure, 260 °C, the mechanism is one of brooming (Figure 1c) due to the significant loss of resin between fibers and the degradation of interfaces resulting in a lack of support for the fibers. The shift in mechanisms from one where there is a bond, even if partial as in the case of splitting failure, to one dominated by brooming and the separation of fibers is expected to result in a decrease in the tensile characteristics of the material, especially at the two highest temperatures of 232 °C and 260 °C, where the matrix and fiber-matrix bond are seen to degrade as can be seen in Figure 2a–c. Figure 2a depicts the material after 4 h where a good bond between the fibers and surrounding resin and plasticity in the resin can be seen. Fiber surfaces that have been exposed due to fracture show pieces of resin still bonded onto them, and with the exception of the fiber at the lower right-hand corner, which shows a crescent-shaped fracture around a part of the fiber with debris in the crack, there is no evidence of debonding or clean pull-out of fibers. In comparison, the initiation of the matrix deterioration around fibers at 16 h of exposure can clearly be seen in Figure 2b wherein gaps can be seen between fibers, and the surrounding resin and fiber surfaces are relatively clean without resin adhering to them in the areas where pull-out has occurred. Figure 2c, in contrast, taken after 48 h of exposure, shows areas with greater levels of large-scale fiber-matrix debonding as well as embrittlement and the deterioration of the resin due to heat as evidenced by the agglomeration of resin in areas both adjacent to the fibers and in the resin-rich zones. The fibers themselves show minor effects of heat at the highest level of exposure. As seen in Figure 3a,b, exposure to elevated temperatures, as expected, initially results in a progression in post-cure causing an increase in tensile strength and modulus. This is in line with earlier results [1,19,45]. It should be noted that after the initial regime that is dominated by post-cure, further exposure results in competition between the mechanisms of the post-cure and degradation of the resin and the fiber-matrix bond. As seen in Figure 3a, post-cure effects, as seen through increases in tensile strength, are noted up to about 16 h, although most specimens reach a maximum by the 8 h level. The highest level of post-cure is noted through exposure at 149 °C for one hour with the tensile strength increasing by 54% above the unexposed levels. This is, however, followed by a decrease that is linear until the end of the 48-h period of exposure. The next highest level of post-cure is seen with the exposure at 66 °C within the 4–16-h time frame. In this case, however, the post-peak drop is greater than at 149 degrees, 23.85% versus 18.14%, as seen in Table 1. The average increase in tensile strength due to exposure from 93 °C onwards, with the exception of the 149 °C regime, is 33.29%, with a standard deviation of 3.83% indicating consistent changes due to postcure, albeit over different time frames. The deteriorative effect of elevated temperature regimes is clearly seen in the case of the 260 °C exposure, where, after a peak in 4 h, there is a steep drop of about 47.6% until the 16-h level, after which there is a slower change of 2.9% per hour until the 72-h level. At this point the residual strength is noted to have decreased by 71.76% from its peak at that temperature. It is noted from Table 1 that the competing effects cause a gradual decrease in the percentage drop from the peak with an increase in temperature from a 20.52% decrease at 121 °C to 11.25% at 232 °C after 72 h of exposure. Assuming that the values in Table 1 are representative of the aggregate (since the two competing effects cannot be completely separated) increase in performance due to post-cure and the aggregate decrease in performance due to deterioration at the level of fiber-matrix bond and in the bulk resin, the trends shown by the two can be assumed to be indicative of the two effects. After the maximum gain from post-cure at 149 °C, the level of increase decreases with the temperature of aging for all except the highest temperature of exposure, indicating the increasing effect of deteriorative mechanisms. However, the overall level for all, with the exception of the specimens exposed to 260 °C, is still positive, indicating a net gain in tensile strength due to postcure.

Hawileh et al. [44] conducted tensile tests on single-layer CFRP composites exposed to temperatures between 25 °C and 300 °C for 45 min after curing for a week and noted 14%, 42%, and 48% decreases in residual strength at 100 °C, 250 °C, and 300 °C, respectively. In the current investigation, at comparable levels of 93 °C and 260 °C after 1 h of exposure, the residual strengths were noted to increase by 25.9% and 24.9%, respectively, due to post-cure effects. In comparison, Foster and Bisby [46] reported decreases of about 20% in residual strength for temperatures up to 300 °C and 80% at 400 °C with Young’s modulus decreasing by less than 10% up to 400 °C. The constant temperature exposures were, however, for extremely short periods of 3 h. At 260 °C, the highest temperature regime considered in the current study, the strengths and moduli did not decrease until the 16-h level of exposure. In comparison, based on a wet layup and room temperature cure carbon-epoxy system, Cao et al. [47] reported an initial decrease of about 31% at temperatures between 40 and 60 °C, followed by a stable response between 60 and 250 °C, with further decrease until 61% beyond 400 °C exposure. The initial drop was explained as being due to a reduction in the shear modulus of the resin because of the initial exposure to elevated temperature levels, with the larger drop at the higher extreme being due to the oxidation of fibers resulting in both fiber and fiber-matrix bond degradation. Given that the level of post-cure possible, and the resulting increases in specific performance characteristics, depends intrinsically on the details of the resin formulation and stoichiometry, process conditions, and the level of conditioning prior to subsequent exposure/aging, it is critical that the conditions and effects are comprehensively assessed, and that databases used for design and analysis are appropriately structured, since otherwise erroneous conclusions could be drawn from short-term exposure results.

As seen from Table 1 and Figure 3b, the tensile modulus also increases initially due to the post-cure effects with the maximum gain in the tensile modulus due to the postcure being seen after 16 h of exposure at 66 °C. Nguyen et al. [36] reported a 7% increase in modulus resulting from 60 min exposure to 200 °C followed by a lower increase at 400 °C and subsequent decreases for pultruded carbon fiber composites. It should be noted that in comparison to the significant increase in tensile strength in the initial stages of exposure to the highest temperature level of 260 °C, the increase in modulus was only 13.24%. This reflects the matrix deterioration-based failure mechanism which causes the fibers to be unsupported at this temperature, allowing fibers to be misaligned, resulting in decreases in moduli. It is important to note that the normal progression of the cure over a 72-h period resulted in a 14.61% increase in the tensile modulus, and thus the maximum increase at 260 °C of 13.24% is essentially a drop from that of the unexposed value over the same period of time, indicating the significant effect of heat on bond deterioration at that combination of temperature-time exposure. A comparison of Figure 3a,b shows that after reaching a peak, the drop in the modulus is fastest between 4 h and 16 h of exposure, similar to that in strength followed by a slower decrease to the final value at 72 h. While the effects of thermal aging on tensile strength have been determined under specific conditions, the effect on the modulus is not as well understood because of the competing effects of post-cure and the progression of polymerization on increasing brittleness which increases the modulus and the increased tendency for microcracking, which decreases it [48]. The current study clearly differentiates between the ranges of the dominance of the two phenomena.

#### 3.1.2. Off-Axis Shear

**Figure 4:**
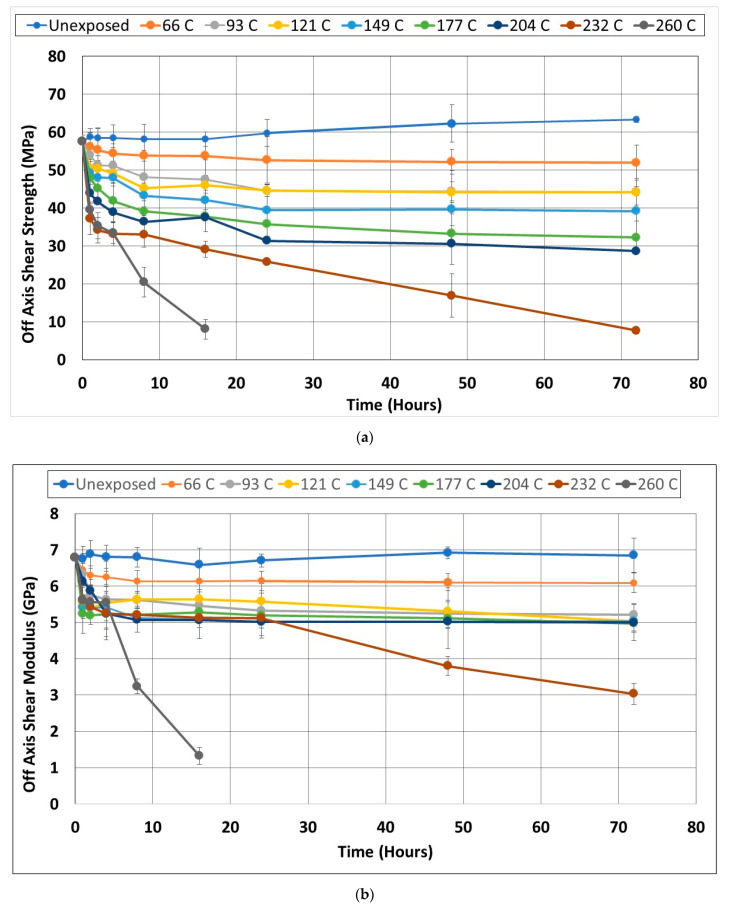
(**a**) Change in Off-Axis Shear Strength as a function of exposure temperature and time; (**b**) Change in Off-Axis Shear Modulus as a function of exposure temperature and time.

**Figure 5:**
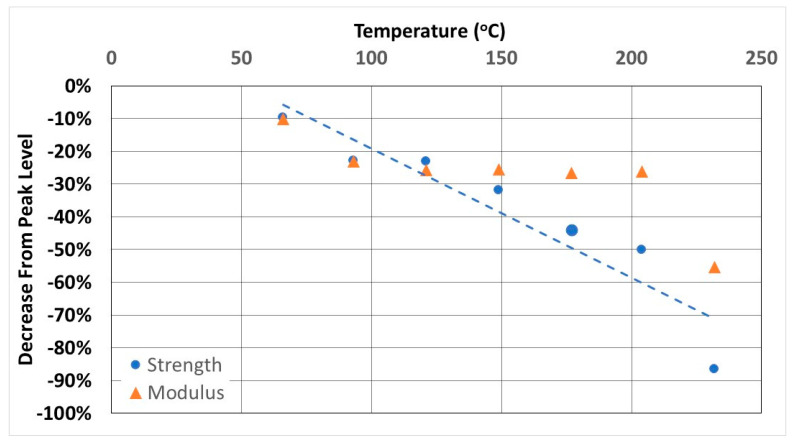
Post peak decrease in Off-axis Shear Strength and Modulus as a function of temperature after 72 h exposure.

While unidirectional tests are effective in assessing fiber-dominated characteristics, the performance due to torsion and shear is perhaps better assessed at the coupon level through off-axis shear tests using a ± θ laminate. This configuration has been shown to yield a high value of shear strain at failure with the shear stress being the major contributor to fractures based on the combined stress failure criteria [49]. Pindera and Herakovich demonstrated that the ±45 configuration, which is used in the current study, yields an accurate determination of shear response with negligible end effects [50]. As seen in Figure 4a,b there is no discernible increase in strength or modulus due to the post-cure accruing from the exposure to elevated temperature. This is due to the immediate weakening effect of temperature on the resin with fibers being at ±45 to the load axis, and thus any softening of the resin causes the separation of layers and the sliding of fibers, whereas increased cure results in brittleness causing matrix failure in the bulk parallel to fibers. With the exception of the unexposed specimens, which do show a minor increase in strength over time, indicative of post-cure under ambient conditions, all other exposures result in a decrease from the initial value with the greatest rate of decrease being in the first 4 h. In the cases of exposures from 66 °C onwards, the level of deterioration in off-axis strength increases with temperature in a linear fashion as shown in Figure 5. The trend for the modulus, however, is different with the level of decrease being almost constant, in a narrow range between 23.2 and 26.7% for temperature regimes between 93 °C and 204 °C, which is an important consideration for design thresholds, especially given the significant drop in both modulus and strength at the higher temperature levels. It is noted that the range within which the performance levels were about constant after the initial drop overlap with that mentioned by Cao et al. [47].

**Figure 6:**
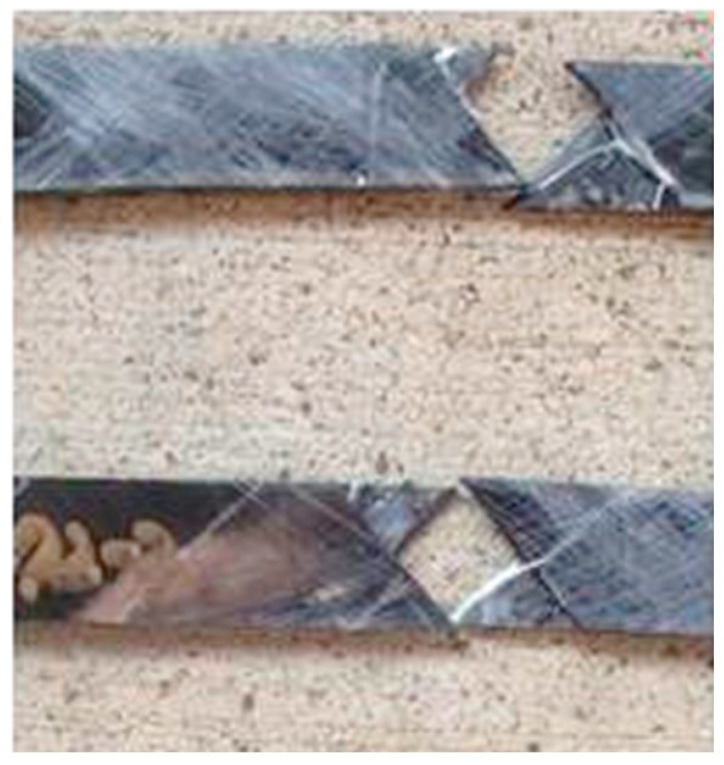
Characteristic failure of off-axis shear specimens.

As seen in Figure 6, fracture occurs parallel to the fiber direction in each layer at temperatures of exposure at, and above, 149 °C. Since the layup has a ±45° configuration, the fracture surfaces are along these angles with the separation of layers between the fracture surfaces demonstrating both fractures in the resin between fibers in a single layer and inter-layer separation in the bulk resin between fabric layers. Due to the use of a manual, rather than an autoclave or high compaction pressure-based, process for the fabrication of the composites, the compaction of layers which would result in the intermingling of fibers from adjacent fabric layers is low, leading to fairly clean inter-layer separation akin to that of the delamination between layers of off-axis prepreg. Off-axis shear specimens subject to higher temperatures of exposure showed distortion and twisting on removal from the oven during cool-down to ambient levels, with this distortion having, as expected, a greater effect on the strength than the modulus because of delamination between the two layers. At the highest temperature of exposure, 260 °C, the distortion is clearly noted after 8 h of exposure, with the level of distortion and delamination increasing at periods of 24 h, and above, to an extent that testing could not be conducted appropriately. While the use of composites for external strengthening of concrete is primarily through the use of unidirectional materials, strengthening for the purposes of shear and torsion as well the use of composites in structural components that are subject to shear incorporate ±45 type architectures. These, as described, are likely to be impacted by the changes seen in exposure to high temperature levels and extended periods of exposure. This is an area without significant study to date and represents an area of future need, especially relating to failure mechanisms and the changing values of shear strength in regions where the shear modulus remains constant within scatter bounds, or decreases very slowly, resulting from changes in the levels of strain.

#### 3.1.3. Flexure

**Figure 7:**
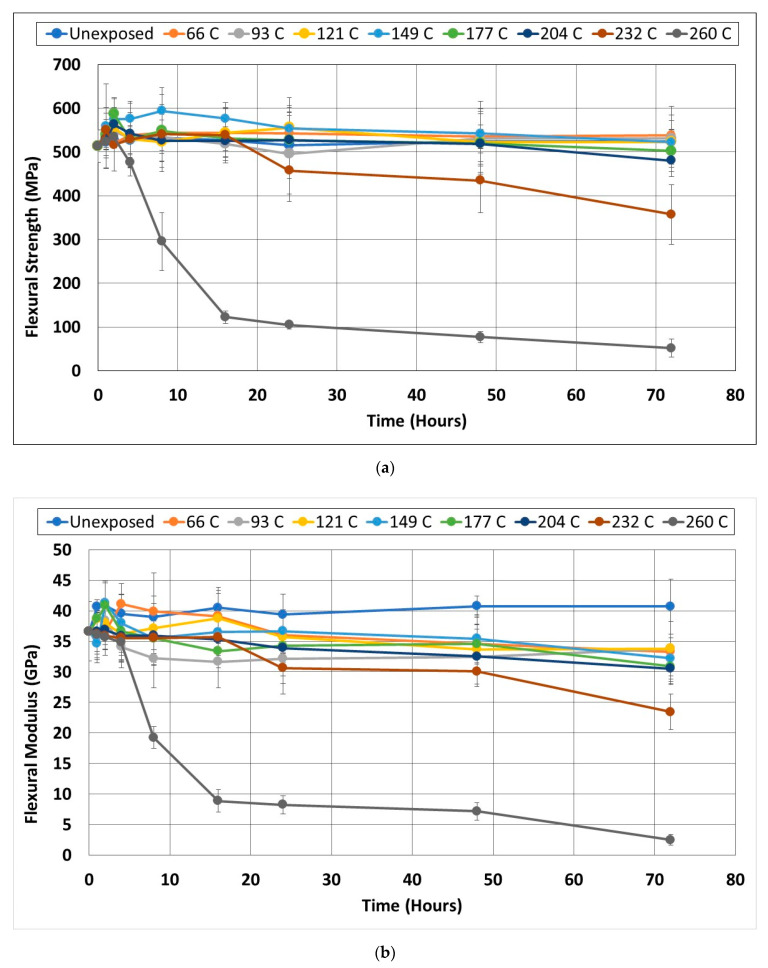
(**a**) Change in Flexural Strength as a function of exposure temperature and time; (**b**) Change in Flexural modulus as a function of exposure temperature and time.

Similar to the off-axis shear characteristics, flexural characteristics are not considered, often for purposes of strengthening, but are of importance in structural components and when the element may itself undergo flexural loading. Since FRP materials are intrinsically layered, they depend on the resin between layers to maintain the integrity of the sample. Under flexural loading, these interfaces are stressed, and this often results in premature failure through excessive deformation or through delamination and interlayer separation induced by the degradation of the resin by thermal exposure even prior to residual testing. It is thus important to assess the effects of prolonged periods of thermal exposure, especially at temperatures significantly in excess of the glass transition temperature, T_g_, in the flexural mode. Overall changes in flexural characteristics as a function of temperature and the time of exposure are shown in Figure 7a,b. An important observation is that unlike in the other tests, the flexural characteristics of strength and modulus showed a decrease below that of the unexposed baseline at all temperatures for modulus, and at temperatures above 177 °C for strength, indicating the criticality of this mode. The maximum increase in flexural strength, 11.7%, above that of the unexposed specimens, is seen after 8 h of aging at 149 °C, which is in the range of increases noted by Garcia-Moreno et al. [48] of 15.3% after 10 days at 150 °C for a quasi-isotropic layup. It is of interest that in that study, the modulus was noted to decrease by 32.5% under that condition whereas in the current study the decrease is lower, at 21.2%, at the end of the longest period of thermal aging of 72 h, i.e., 3 days at 149 °C. Since the integrity of interlayer bonds is essential for the flexural response, degradation at this level results in the consistent reduction in a flexural response below that of the unexposed specimens overcoming the initial positive effects of the post-cure. At all levels in the current study, decreases were due to microcracking in resin interlayers and due to the degradation of the fiber-matrix bond. These failure mechanisms are fairly local and are significantly affected by aspects such as voids, fiber misalignment, and resin-rich areas, especially between layers of fabric, resulting in larger levels of standard deviation for these results as compared to the others.

It should be noted, however, that the level of 80% decrease in flexural strength noted by Sorathia et al. [31] is not reached until 24 h of exposure at 260 °C. Vieira et al. [22] exposed pultruded glass fiber reinforced polymer composites to temperatures between 120 °C and 320 °C for 30 min after which the specimens were tested in flexure. Due to the presence of significant defects and porosity, their results showed considerable non-uniform (random increases and decreases across the temperature range) trends with temperature. However, the maximum decreases for the isophthalic polyester and vinylester-based composites were noted after exposure to 170 °C to be 25.6% and 18.9%, respectively, with a second vinylester system showing a 7.3% decrease at 220 °C. While direct comparisons cannot be drawn with the current study due to differences in resin and fiber, it is worth noting that at 177 °C the carbon-epoxy specimens only show a 2% decrease in strength after 72 h of exposure and a 10.8% decrease after 24 h at 232 °C. In comparison, Garcia-Moreno et al. [48] reported decreases of 42.4% and 40.8% in flexural strength and modulus, respectively, after 10 days at 230 °C, and 73.3% and 43.5%, respectively, after 10 days at 250 °C. The effect of the quasi-isotropic layup in contrast to the unidirectional used herein needs, however, to be kept in mind given the predominance of interlayer separation-based mechanisms which are increased with layers at different orientations.

As in the case of tensile strength, the maximum increase in flexural strength due to the post-cure was due to exposure at 149 °C, but the level of increase is significantly lower. The drop in post-peak strength is seen to increase with temperature with the largest drop of 90.33% being after 72 h of exposure at 260 °C. The most significant decreases are from the 48-h period onwards with drops of 3.3% to 3.75% at 149 °C and 177 °C followed by 74% at 204 °C, 17.8% at 232 °C, and 32.8% at 260 °C. The overall response for exposure beyond 48 h appears to be very similar within scatter bounds for all specimens exposed to temperatures between 66 °C and 149 °C, with additional decreases at higher temperatures, suggesting a change in the failure mode at the higher temperatures. For the temperature at which the most significant decreases were seen, 260 °C, the drop occurs extremely steeply between 4 and 16 h at a rate of about 30% per hour which is higher than that noted for tensile strength at 26.5% per hour over the same time period. Beyond this period from 16 h to 72 h, the drop is significantly lower in both cases at 2.88% per hour and 1.26% per hour for flexural strength and tensile strength, respectively. As seen in Figure 7b there are significantly greater decreases in post-peak flexural modulus values than in flexural strength, although the increases due to the post-cure are lower except for the unexposed and 66 °C exposure conditions. In this context, it is important to note that while fractures were noted on the tensile face for specimens exposed to temperature regimes up to 149 °C, higher temperatures resulted in a transition to an interlaminar failure mode through the delamination/separation of layers for specimens with the mode being dominant at periods of exposure greater than 24 h.

#### 3.1.4. Short Beam Shear

**Figure 8:**
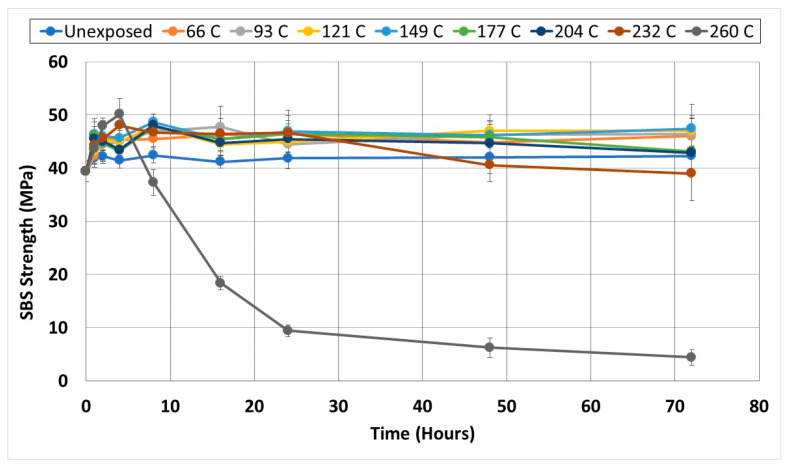
Change in short beam shear strength as a function of exposure temperature and time.

**Figure 9:**
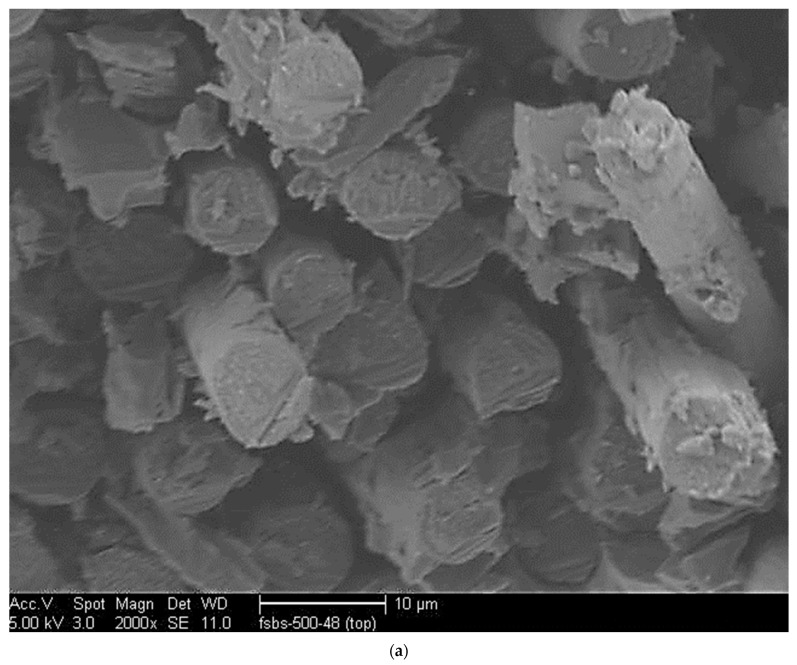

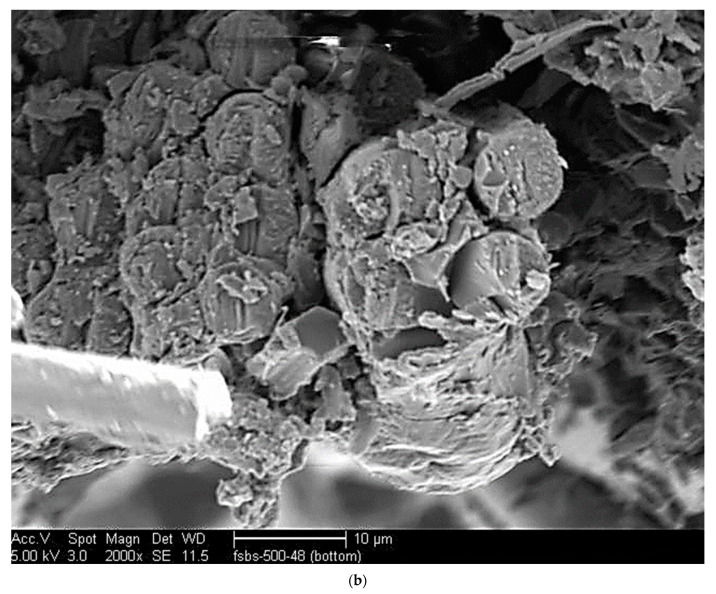
(**a**) SEM Image of SBS specimen near the top surface after testing after 48 h of exposure at 260 °C; (**b**) SEM Image of SBS specimen near the top surface after testing after 48 h of exposure at 260 °C.

Given the criticality of the interfacial region, it is useful to also assess residual mechanical behavior in SBS specimens which are often used as a means of material screening and assessment of quality control due to their simplicity. As seen in Figure 8, at all exposure levels, except the two highest, the final SBS strength is higher than the initial unexposed level indicating the dominance of the post-cure effect over that of the deterioration within these regimes. It is of interest to note that while the unexposed specimens increased in SBS strength by 7.55% due to the 72-h exposure, exposures between 93 °C and 232 °C showed an increase between approximately 21% and 23.5% which, when viewed in terms of the scatter band, should be considered as similar. At 66 °C the increase is slightly lower at 17.7% and at 260 °C it is higher at 27%. As with all other tests, the maximum degradation was seen after exposure to the 260 °C temperature regime with decreases occurring within three distinct regimes of 4–8 h, 8–24 h, and 24–72 h with the rates of decrease of 11.4% per hour, 4% per hour, and 1.1% per hour, respectively. The trend of an initial increase in SBS strength followed by a decrease over longer periods of exposure follows results reported by Zavatta et al. [42] where there was insignificant deterioration up to 198 °C and then very steep decreases at 260 °C, which was noted as being 145% of the T_g_ of 71% and 89% at the 24 h and 72 h levels of exposure, respectively. Their results are very similar to those from the current study of 76% at 24 h and 89% at 72 h. It is seen that interlaminar failure is the dominant mode initially, transitioning through greater matrix degradation and failure to one of fiber-matrix debonding and interlayer separation as shown in Figure 9 for specimens representative of thermal aging at 232 °C for 48 h and above and at 260 °C at 8 h and above. Both these images show debonds between the fiber and matrix, with fiber surfaces in the pull-out areas, as seen in Figure 9a, not having a significant matrix adhering to them. The transition in the mode where fiber pullout and debonding become dominant was also noted by Akay et al. [51]. In all cases except at the highest temperature and only after 48 h of exposure, the failure modes were as expected for short beam shear with good bonding between layers.

### 3.2. Thermal Analysis

**Figure 10:**
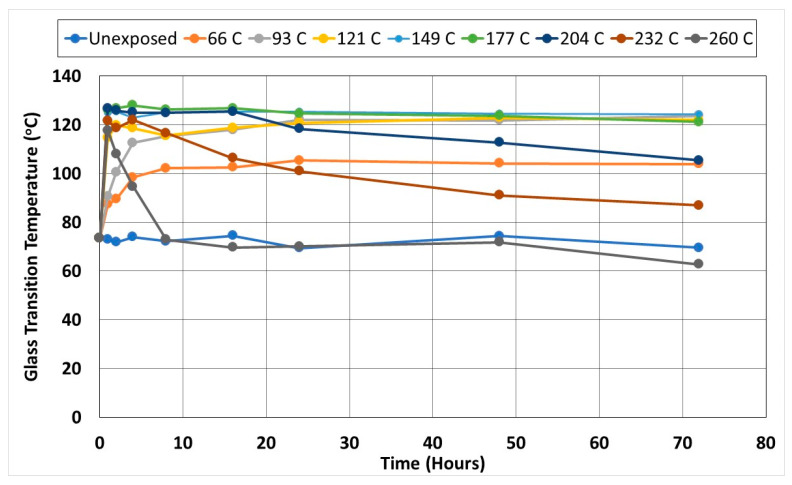
Change in DSC derived glass transition temperature as a function of exposure temperature and time.

For the purposes of the current investigation, DSC characterization was used to assess the relative levels of post-cure attained through exposure to temperature regimes and levels of the glass transition temperature, which was determined from the midpoint of the inclined part of the heat flow curve. It is noted that the use of DSC results in the determination of a value for the glass transition temperature lower than that from Dynamic Mechanical Analysis (DMA) and Thermo-Mechanical Analysis (TMA) is expected to be the most precise [52,53]. Given the effect of thermal aging on the post-cure and hence on an increase in T_g_, this method was deemed to be more suitable for the current investigation. As seen in Figure 10, there is a significant gain in T_g_ from the initial value, with peaks being attained after one hour of exposure at temperatures of 149 °C, after four hours at 121 °C, and after 24 h at the two lowest temperatures of 66 °C and 93 °C. The maximum level of T_g_ attained between 125.5 °C and 128 °C, representing an increase of 74.5% above the initial unexposed level of 73.35 °C, relates to thermal aging at temperatures of 149 °C, 177 °C, and 204 °C. Aging at temperatures between 66 °C and 149 °C shows very minor deterioration from the peak level, and all tend to the same final level. Exposure to 177 °C results in a slight decrease after 24 h which correlates with the earlier noted initiation of deterioration in the resin-dominated mechanical characteristics. Exposure to higher temperature levels results in more rapid decreases from the 16 h level at 204 °C, the 4 h level at 232 °C, and the 1 h level immediately post-peak at the highest temperature of exposure of 260 °C, wherein the major deterioration is noted between 1 and 8 h (a 38.1% decrease, after which further changes are much smaller).

While one might want to see a direct correlation between the changes in T_g_ and those in mechanical characteristics, it should be remembered that the glass transition temperature is not, as such, a discreet event but is rather a thermal transition over a range of temperatures. As related to polymers and composites, it represents the change from a “glassy” to a “rubbery” state and is associated with changes in strength, toughness, and stiffness. Thus, the attainment of the peak in T_g_ should not, in itself, be thought of as the point of a sudden change in mechanical characteristics but as an indicator of transition. In this vein, the decreases in T_g_ in Figure 10 can be related to the deterioration of the polymer network, matrix microcracking, and fiber-matrix debonding, all of which were noted through microscopy and changes in response in the mechanical characterization tests. As described earlier, significant distortion of the off-axis specimens is noted initiating at the 8 h level under conditions of thermal aging at 260 °C which interestingly is the point that indicates the end of a significant drop in glass transition temperature from the peak transitioning to a very slow further decrease.

Further characterization through dynamic mechanical thermal analysis (DMTA) and thermogravimetric analysis (TGA) methods to correlate changes in the glass transition temperature with energetic and modulus changes, as well as those in the loss modulus peak, are needed to study the initiation and onset of deterioration and the transition more deeply from the post-cure to thermal aging-based degradation and will be reported on separately.

## 4. Discussion and Conclusions

The current study provides a comprehensive investigation of the residual mechanical characteristics of ambient cured wet layup carbon-epoxy composites over a range of temperatures between ambient (23 °C) and 260 °C for periods of time between 1 and 72 h. This provides an extensive dataset that is of value in understanding the changes in mechanisms of failure with increases in time and temperature of exposure, as well as providing post-exposure mechanical characteristics of use in the design and assessment of structural resilience and integrity. It is seen that thermal aging initially causes competition between the mechanisms of post-cure, which can result in significant increases in fiber-dominated mechanical properties and heat-related deterioration that negatively affects both the resin and the fiber-matrix bond. The tensile strength and modulus are seen to not be negatively affected until 16 h of exposure at the highest temperature level of 260 °C, emphasizing that composites used primarily in the tension mode can withstand fairly long periods of elevated temperature exposure without seeing decreases in performance of fiber dominated characteristics. In comparison, the off-axis characteristics that are resin- and interface-dominated are affected at lower temperatures of aging and short periods of exposure, although the drops are not significant until the longer period and higher temperature combinations occur. Flexural strength is seen to show decreases after 72 h at 177 °C with a change in the failure mode beyond this regime. In contrast, the flexural modulus is seen to decrease at fairly low temperatures due to initial interlaminar failure modes that cause delamination/separation/splitting between layers.

While the understanding of effects by themselves and of the progression of changes in materials’ characteristics as a function of thermal aging as investigated in this study is important, as discussed by Baghad et al. [54], it may also be of value from the perspective of materials’ use and design to understand the relative effects of the two major factors under consideration—operating temperature and holding time at that temperature. Their results on a quasi-isotropic autoclave-cured laminate indicated that operating temperatures dominated the change in mechanical properties due to thermal aging. Using their approach of averaging across the extremes and assessing the differences between the maximum and minimum values of residual performance for all combinations except for the highest temperature of 260 °C, where very large drops were noted after a threshold of time, it is seen that, with the exception of tensile strength, off-axis shear modulus, and flexural strength, where the differences where the temperature of exposure had a slightly higher effect that the hold time (35.14% versus 33.15% for tensile strength, 34.72% versus 31.39% for off-axis shear modulus, and 12.95% versus 10.31% for flexural strength), all others were dominated by the effects of holding time (26.56% versus 21.38% for tensile modulus, 47.65% versus 9.47% for off-axis shear strength, 19.39% versus 9.61% for flexural modulus, and 18.50% versus 9.99% for short beam shear strength). While it needs to be emphasized that these observations must be viewed in terms of extremes, i.e., maximum and minimum residual performance characteristics attained across a factor, rather than in isolation, it does suggest that for specific characteristics the time of exposure may have a significantly greater deteriorative effect than the actual temperature for the ranges considered in this study. This is of importance in viewing the effects of aging parameters on the post-exposure risk and integrity of the structure, especially in terms of short-term post-event operation.

The differences in deterioration between fiber, resin, and interlayer-dominated mechanical characteristics as shown through this investigation reinforce the importance of assessing other mechanical characteristics in addition to tension as is performed currently, since the lack of that information could lead to inadvertent failure in a structure due to other modes of loading, even if minor.

## Figures and Tables

**Table 1:** Percentage Change in Characteristics.

	Unexposed	66 °C	93 °C	121 °C	149 °C	177 °C	204 °C	232 °C	260 °C
**Tensile Strength**									
**Increase from Initial**	5.42%	33.91%	37.66%	31.83%	54.42%	36.37%	34.12%	26.82%	32.95%
**Decrease from Peak**	0.00%	−23.85%	−12.89%	−20.52%	−18.14%	−17.67%	−13.53%	−11.25%	−71.76%
**Tensile Modulus**									
**Increase from Initial**	14.61%	58.59%	42.70%	20.53%	33.34%	27.94%	29.88%	30.91%	13.24%
**Decrease from Peak**	0.00%	−24.00%	−23.08%	−21.54%	−14.80%	−17.26%	−10.69%	−20.83%	−69.91%

## Data Availability

The data presented in this study are available on request from the corresponding author.
